# Impact of ascending aortic length to detect surgical intervention for ascending aortic aneurysms

**DOI:** 10.1007/s11748-025-02176-5

**Published:** 2025-06-25

**Authors:** Toshikuni Yamamoto, Akihiko Usui, Tomonari Uemura, Ryota Yamamoto, Hideki Ito, Tomo Yoshizumi, Sachie Terazawa, Yoshiyuki Tokuda, Yuji Narita, Masato Mutsuga

**Affiliations:** 1 Department of Cardiac Surgery, Nagoya University Graduate School of Medicine, 65 Tsurumai, Showa-Ku, Nagoya, Aichi 466-8550 Japan; 2 Department of Cardiovascular Surgery, Fujita Health University Okazaki Medical Center, Okazaki, Aichi Japan

**Keywords:** Ascending aortic aneurysm, Ascending aortic length, Length height index

## Abstract

**Objective:**

Ascending aortic length (AAL) has recently garnered attention as an additional parameter of surgical indication. This study aimed to verify that AAL is extended in ascending aortic aneurysm patients when compared with the normal aorta.

**Methods:**

The study included 132 patients who were diagnosed with true ascending aortic aneurysms from January 2002 to December 2021. The AAL was measured as the distance from the aortic annulus to the origin of the innominate artery. The data of 295 patients who underwent transcatheter aortic valve replacement during same period were compiled as the control group. In order to index AAL, it was divided by the patient’s height (Length height index, LHI).

**Results:**

The mean ascending aortic diameter (AAD) and AAL in the 132 patients were 5.3 ± 0.6 cm and 11.7 ± 1.6 cm, respectively. Propensity score matching revealed a significantly longer AAL in the aortic aneurysm group than in the control group (11.7 vs. 8.8 cm, *P* < 0.05). The LHI in the aortic aneurysm group was significantly greater than in the control group (7.4 vs. 5.7 cm/m, *P* < 0.05). The relationship between AAD and LHI was analyzed using linear regression analysis. The regression coefficient was 0.59, and the intercept was 4.22. As a tool to predict LHI, the formula: LHI = 0.59 × AAD + 4.22 was obtained.

**Conclusions:**

AAL and LHI were significantly increased in patients with ascending aortic aneurysms. Consequently, LHI may serve as an accurate indicator of surgical intervention.

**Graphical Abstract:**

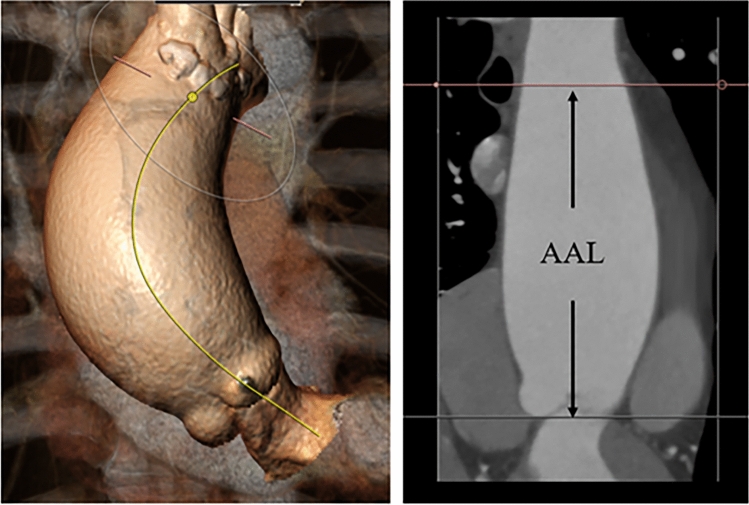

## Introduction

Ascending aortic aneurysm patients are at elevated risk for adverse aortic events (AAEs). To prevent these complications, current clinical guidelines recommend the elective repair of ascending aortic aneurysms with diameters exceeding 5.5 cm [1–5]. However, ascending aortic diameter (AAD) alone cannot accurately stratify risk [6]. Data from International Registry of Acute Aortic Dissections revealed that employing an AAD cutoff of > 5.5 cm fails to identify 60% of type A aortic dissection patients [7]. Therefore, ascending aortic length (AAL) has recently garnered attention as an additional parameter of surgical indication. The Yale group proposed that an AAL > 11 cm be established as a threshold for intervention [8]. They proposed that an AAL of 11 cm was more strongly associated with long-term AAEs. In addition, they indexed AAL by patient height (length-height index [LHI] = AAL/height). This study aimed to verify that AAL is extended in ascending aortic aneurysm patients when compared with the normal aorta. In addition, we investigated the association between AAD and LHI and evaluated its potential applications as a surgical intervention index.

## Methods

### Patients

The study included 132 patients who were diagnosed with true ascending aortic aneurysms (aortic aneurysm group) among the 1245 patients who underwent ascending aortic replacement at our institution from January 2002 to December 2021. The exclusion criteria included the presence of aortic dissection, pseudoaneurysm, isolated aortic root aneurysm, isolated aortic arch aneurysm, and Marfan syndrome. Patients in whom contrast-enhanced computed tomography (CT) was not performed were excluded. The baseline data were retrieved from physician reports and medical records. This study was approved by our institutional review board, which waived the need for individual patient consent (No. 2022–29). To investigate an aortic elongation of ascending aortic aneurysm, the data of 295 patients who underwent transcatheter aortic valve replacement (TAVR) during same period were compiled (control group). Using propensity score matching, 132 matched pairs were generated.

### Measurement

**Fig. 1:**
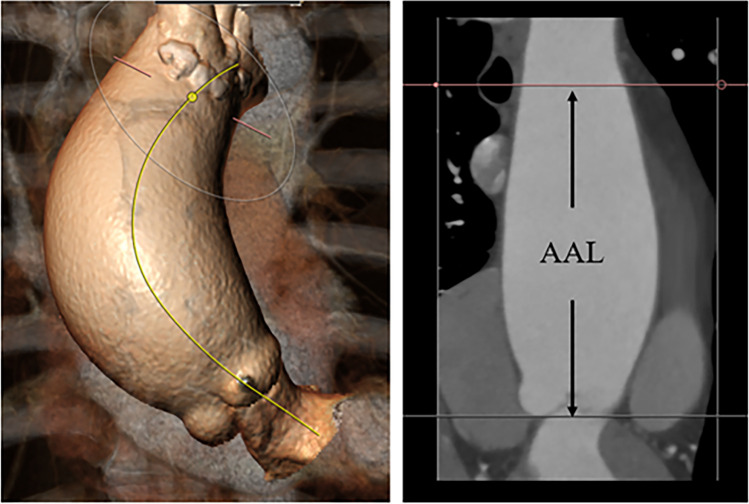
The AAL was measured as the distance from the aortic annulus to the origin of the innominate artery using the 3mensio software

The contrast-enhanced CT data were analyzed using the 3mensio software (Pie Medical Imaging, The Netherlands). The AAL was measured as the distance from the aortic annulus to the origin of the innominate artery using the center-line method (Fig. 1). In addition, LHI was defined as AAL divided by the patient’s height.

### Statistical analysis

Categorical variables were reported as absolute and relative frequencies. Continuous variables were reported as means and standard deviations or as medians and interquartile ranges. The AAL and LHI between-group differences were compared using T tests. A *p* value of < 0.05 was considered to be statistically significant. Linear regression was used to estimate the relationship between AAD and LHI. The statistical analysis was performed using the EZR software (Saitama Medical Center, Jichi Medical University, Saitama, Japan) [9], which is a graphical user interface of R (The R Foundation for Statistical Computing, Vienna, Austria). It is a modified version of R Commander that incorporates frequently employed biostatistics.

## Results

### Patient baseline characteristics

Table 1 provides a summary of the patients’ baseline characteristics. The mean age of the study participants was 67.2 ± 11.3 years, and 54.5% were men. Hypertension was present in 68 patients (51.5%), renal failure in 43 (32.6%), chronic obstructive pulmonary disease in 10 (7.6%), and peripheral artery disease in 27 (29.5%) patients. Bicuspid aortic valve (BAV) was present in 48 patients (36.4%). Thirty-three patients (25.0%) had undergone concomitant aortic root replacement, and 69 patients (52.3%) underwent aortic arch replacement.

**Table 1:** Patient baseline characteristics

	Values (*n* = 132)
Age, years, mean ± SD	67.2 ± 11.3
BSA, cm/m^2^	1.6
Male	72 (54.5%)
Hypertension	68 (51.5%)
Dyslipidemia	43 (32.6%)
Diabetes mellitus	15 (11.4%)
Renal failure	43 (32.6%)
Chronic obstructive pulmonary disease	10 (7.6%)
Cerebral vascular accident	12 (9.1%)
Peripheral artery disease	27 (20.5%)
Cardiac dysfunction	8 (6.1%)
Smoking	57 (43.2%)
Angina pectoris	19 (14.4%)
Aortic stenosis	37 (28.0%)
Aortic regurgitation	38 (28.8%)
Bicuspid aortic valve	48 (36.4%)
Takayasu arteritis	10 (7.6%)
Aortic root aneurysm	33 (25.0%)
Aortic arch aneurysm	69 (52.3%)

### AAL and LHI

**Fig. 2:**
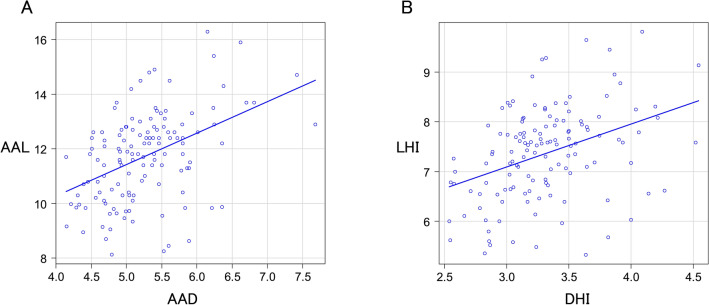
A positive correlation was observed between AAD and AAL (**A**) and between DHI and LHI (**B**)

**Fig. 3:**
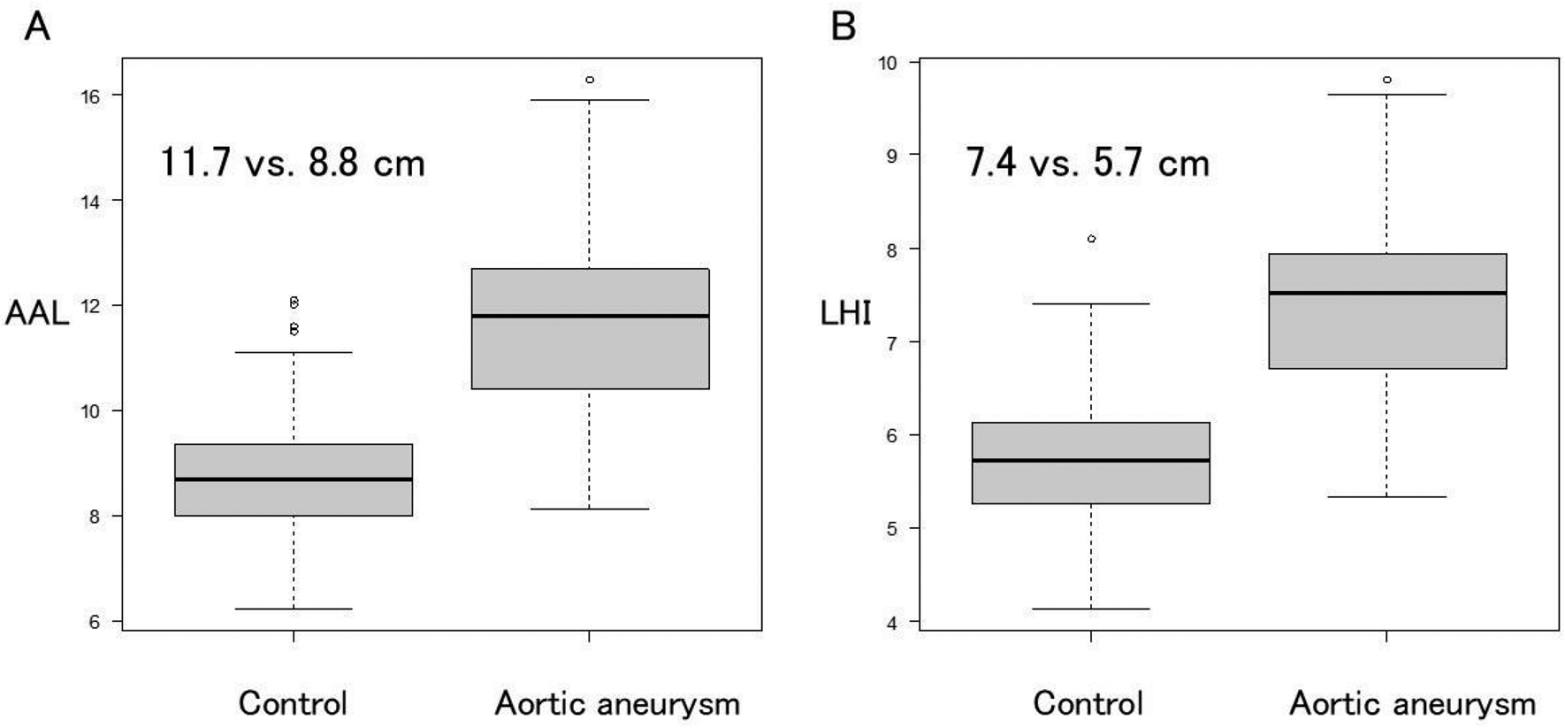
The AAL in the aortic aneurysm group was significantly longer than in the control group (**A**). The LHI in the aortic aneurysm group was significantly greater than in the control group (**B**)

The mean AAD and AAL in the 132 patients were 5.3 ± 0.6 cm and 11.7 ± 1.6 cm, respectively. The mean diameter height index (DHI) was 3.3 ± 0.4 cm/m, and the LHI was 7.4 ± 0.9 cm/m. A positive correlation was observed between AAD and AAL (regression coefficient = 1.15, *P* < 0.5) (Fig. 2A) and between DHI and LHI (regression coefficient = 0.86, *P* < 0.5) (Fig. 2B). Propensity score matching (Table 2) revealed a significantly longer AAL in the aortic aneurysm group than in the control group (11.7 vs. 8.8 cm, *P* < 0.05) (Fig. 3A). The LHI in the aortic aneurysm group was significantly greater than in the control group (7.4 vs. 5.7 cm/m, *P* < 0.05) (Fig. 3B).

**Table 2:** Propensity score matching

	Aortic aneurysm (*n* = 132)	Control (*n* = 132)	*p* value
Age	67.2	81.8	< 0.001
BSA	1.6	1.5	< 0.001
Male	72 (54.5%)	67 (50.8%)	0.622
Hypertension	68 (51.5%)	77 (58.3%)	0.322
Dyslipidemia	43 (32.6%)	70 (53.0%)	< 0.001
Diabetes mellitus	15 (11.4%)	25 (18.9%)	0.122
Renal failure	43 (32.6%)	69 (52.3%)	0.002
Chronic obstructive pulmonary disease	10 (7.6%)	9 (6.8%)	1.000
Cerebral vascular accident	12 (9.1%)	14 (10.6%)	0.837
Peripheral vascular disease	27 (20.5%)	29 (22.0%)	0.880
Cardiac dysfunction	8 (6.1%)	8 (6.1%)	1.000
Smoking	57 (43.2%)	58 (43.9%)	1.000
Angina pectoris	19 (14.4%)	15 (11.4%)	0.582
Bicuspid aortic valve	48 (36.4%)	16 (12.1%)	< 0.001

### Linear regression analysis

**Fig. 4:**
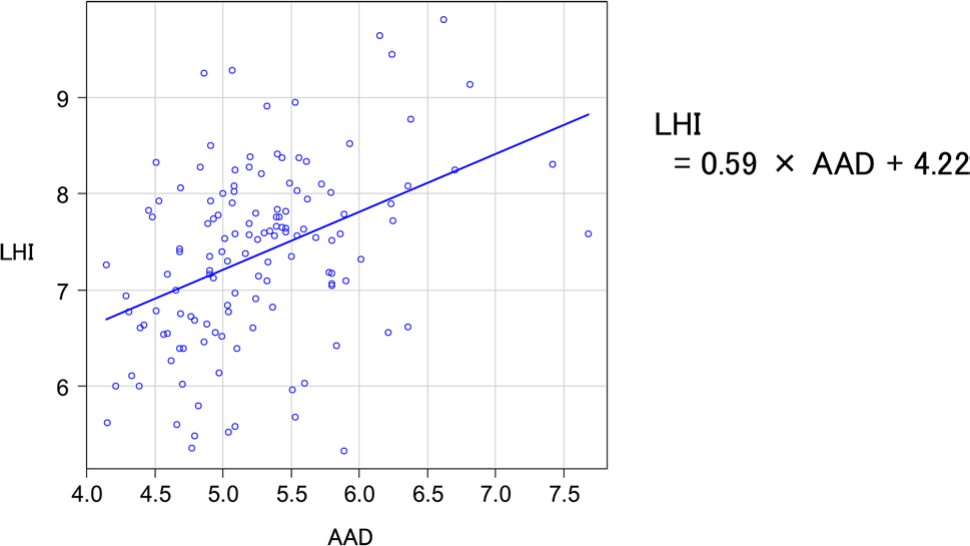
The regression coefficient was 0.59, and the intercept was 4.22

The relationship between AAD and LHI was analyzed using linear regression analysis. A positive correlation was observed between AAD and LHI. The regression coefficient was 0.59, and the intercept was 4.22. As a tool to predict LHI, the formula: LHI = 0.59 × AAD + 4.22 was obtained (Fig. 4).

### Bicuspid aortic valve

**Fig. 5:**
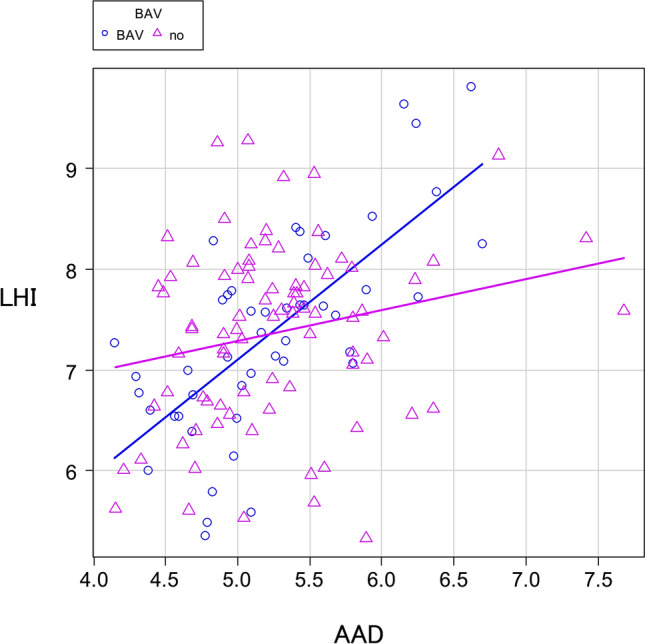
In patients with BAV, linear regression analysis demonstrated a significant trend toward aortic longitudinal elongation compared to non-BAV group

Of the 132 patients, BAV was detected in 48 patients (36.4%). Stratification was performed based on the presence or absence of BAV. In patients with BAV, linear regression analysis demonstrated a significant trend toward aortic longitudinal elongation compared to non-BAV group (regression coefficient: 1.14 vs. 0.31) (Fig. 5).

## Discussion

Krüger and associates published an early investigation into the correlation between classical AAL and AAEs [10]. They discovered that the AAL was increased in patients with acute aortic dissection compared to the healthy controls (108 mm vs. 84 mm, *P* < 0.001). The Yale group has demonstrated that an aortic elongation can result in AAEs and has proposed that an aortic elongation of 11 cm should be considered as a potential intervention criterion for ascending aortic aneurysms [8]. The present study examined patients who underwent aortic aneurysm surgery, and their mean AAD was 5.3 cm and their mean AAL was 11.7 cm. When contrasted with prior research, these figures were considered to be valid. There was a positive correlation between these two figures. Furthermore, we demonstrate that the AAL is extended in patients with ascending aortic aneurysm by comparing it with a normal aorta. In the present study, the AAL in the aortic aneurysm group was significantly longer than that in the control group (11.7 vs. 8.8 cm, *P* < 0.05).

Previous studies proposed the concept of indexing aortic dimension or diameter by patient height to improve the process of surgical decision-making in patients with aortic aneurysms [11–13]. Zafar and colleagues reported that the height-based ratio, which is less complex and does not involve weight and body surface area calculations, yields satisfactory results for evaluating the risk of AAEs in patients with ascending aortic aneurysm [13]. However, these indices never gained widespread clinical use as a marker for aortic intervention. The initial investigation of LHI was conducted by Wu and colleagues [8]. They indexed AAL by patient height. They reported that LHI > 7.5 cm/m was associated with a > fivefold higher average yearly rate of AAEs compared with LHI < 5.5 cm/m. Although the method of dividing AAL by height is not yet widely accepted, it has enabled them to demonstrate the relationship between AAL and AAE. We also adopted this approach. In the present study, LHI in the aortic aneurysm group was significantly higher than LHI in the control group (7.4 vs. 5.7 cm/m, *P* < 0.05). These findings confirmed that aortic aneurysm patients experience longitudinal elongation of the ascending aorta.

The relationship between AAD and LHI was evaluated through a linear regression analysis. The regression coefficient was 0.59, and the intercept was 4.22. This gave us the formula: LHI = 0.59 × AAD + 4.22. Based on this formula, the AAD can be used to produce a predicted value for LHI. If the actual value for LHI exceeds the predicted value, it suggests a strong tendency toward aortic elongation. Although the previous studies revealed the threshold value for LHI, it is doubtful that LHI alone can serve as an indicator of surgical intervention. The AAD is clearly an absolute indicator. Therefore, what is needed are supplementary indicators. We evaluated the LHI on the basis of the actual value for AAD. The actual value for LHI exceeds the predicted value, which can be taken as meaning that the aorta is elongated. Using AAD as an absolute indicator and also taken LHI into account, more accurate assessment can be obtained. Therefore, it was considered useful to use our formula as a surgical intervention indicator.

Approximately, 40% of patients with BAV develop an aneurysm or enlargement of the ascending aorta [14]. It has also been reported that patients with BAV are at a higher risk of developing acute aortic dissection at an earlier age [15]. For these reasons, patients with BAV and ascending aortic aneurysms have been recommended for early aortic treatment [16]. In the present study, 48 patients exhibited BAV. We conducted an additional linear regression analysis and stratified patients based on the presence or absence of BAV. The regression coefficients were 1.14 and 0.31, respectively, and the regression coefficient tended to be higher in patients with BAV. In other words, patients with BAV demonstrated greater ascending aorta elongation. Longitudinal extension of the aorta may be related to a higher incidence of AAEs.

The study had multiple limitations. This included a small number of patients and a retrospective observational design. It is uncertain whether LHI is associated with AAEs, as our study assessed LHI in postoperative patients rather than patients with AAEs. The comparison with patients with a normal aorta was solely undertaken in terms of its length. Furthermore, patients with a normal aorta who underwent TAVR comprised the control group, not noncardiac patients. Considering that 132 patients included patients with aortic regurgitation, it would be a confounding factor that all patients who underwent TAVR had aortic stenosis. TAVR patients were selected as a control group because it was necessary to select patients who had undergone contrast-enhanced CT. For further development of our study, patients without aortic valve disease should be selected.

## Conclusion

AAL and LHI were significantly increased in patients with ascending aortic aneurysms. Consequently, LHI may serve as an accurate indicator of surgical intervention. 

## Data Availability

The datasets used and/or analyzed during the current study are available from the corresponding author on reasonable request.

## Abbreviations

AAE: Adverse aortic event
AAD: Ascending aortic diameter
AAL: Ascending aortic length
LHI: Length height index
CT: Computed tomography
TAVR: Transcatheter aortic valve replacement
BAV: Bicuspid aortic valve
DHI: Diameter height index
BSA: Body surface area
